# Immunomodulatory Effects of a Standardized Botanical Mixture Comprising *Angelica gigas* Roots and *Pueraria lobata* Flowers Through the TLR2/6 Pathway in RAW 264.7 Macrophages and Cyclophosphamide-Induced Immunosuppression Mice

**DOI:** 10.3390/ph18030336

**Published:** 2025-02-27

**Authors:** Seo-Yun Jang, Hyeon-A Song, Min-Ji Park, Kyung-Sook Chung, Jong Kil Lee, Eun Yeong Jang, Eun Mi Sun, Min Cheol Pyo, Kyung-Tae Lee

**Affiliations:** 1 Department of Pharmaceutical Biochemistry, College of Pharmacy, Kyung Hee University, Seoul 02447, Republic of Korea; tjdbs2357@naver.com (S.-Y.J.); sha3076@naver.com (H.-A.S.); mjpark957@naver.com (M.-J.P.); adella76@hanmail.net (K.-S.C.); 2 Department of Fundamental Pharmaceutical Science, Graduate School, Kyung Hee University, Seoul 02447, Republic of Korea; jklee3984@khu.ac.kr; 3 Chong Kun Dang Healthcare Co., Seoul 04300, Republic of Korea; eyjang@chlabs.co.kr; 4 CH Labs Corp., Seoul 07249, Republic of Korea; emsun@chlabs.co.kr (E.M.S.); pminch89@chlabs.co.kr (M.C.P.)

**Keywords:** medicinal herb, immunomodulatory, macrophages, toll-like receptor 2/6, intestine

## Abstract

**Background**: As the population ages, enhancing immune function is crucial to mitigating age-related physiological decline. Since immunostimulant drugs are known to have potential side effects, medicinal plants emerge as promising candidates offering a safer alternative. To leverage the advantages of medicinal plants with fewer side effects and develop a potent immune-enhancing agent, we investigated the efficacy of a novel immunomodulatory candidate derived from the combination of *Angelica gigas* and *Pueraria lobata* (CHL). **Methods**: In vitro, CHL was treated in RAW 264.7 macrophages at various time points, and the experiments conducted in the study were performed using ELISA, Western blot, and RT-qPCR analysis. In vivo, C57BL/6 mice were administrated CHL for 16 days (p.o.) and CTX on the three days (i.p.), and experiments were conducted with ELISA, western blot, RT-qPCR analysis, H&E staining, flow cytometry, gut microbiome, and correlation analysis. **Results**: In vitro, CHL has upregulated NO and cytokines expression, substantially enhancing the NF-κB and MAPK activation. Furthermore, CHL promoted the TAK1, TRAF6, and MyD88 via TLR2/6 signaling. In vivo, the CHL improved the reduced body weight and immune organs’ indices and recovered various cytokines expression, NK cell cytotoxicity activity, and immune cell population. CHL also improved the histological structure and tight junction markers, mucin-2, and TLR2/6 in the intestines of CTX-induced mice. **Conclusions**: Overall, CHL demonstrated immunostimulatory potential by enhancing immune responses and restoring immune function, suggesting its promise as a safe and effective immune-enhancing agent.

## 1. Introduction

The immune system plays a major role in maintaining homeostasis through complex mechanisms involving immune cells. Although the immune system reacts against external invasion, the immune response is easily disrupted by various factors, such as aging, stress, and diseases [1,2]. Dysfunction of the immune response can result in weakened regenerative capacity, chronic inflammation, and downregulated interactions between immune cells. Therefore, as the population continues to age, healthy functional foods have recently been developed to prevent the development of an abnormal immune response [3]. To protect against immune response dysregulation, immune cells produce immunostimulatory mediators, such as nitric oxide (NO), inducible NO synthase (iNOS), tumor necrosis factor-α (TNF-α), interleukin (IL)-6, and IL-1β via activated TNF-induced nuclear factor kappa B (NF-κB) and activator protein-1 (AP-1) signals [4]. The NF-κB and AP-1 pathways are regulated by the phosphorylation of inhibitors of NF-κB (IκB)-α and IκB kinase α/β (IKKα/β)] or the mitogen-activated protein kinase (MAPK) signaling pathways [4], which are modulated by identical upstream signals, [transforming growth factor-β-activated kinase 1 (TAK1)/TNF receptor-associated factor 6 (TRAF6)/myeloid differentiation primary response 88 (MyD88)/toll-like receptor (TLR)] [5].

The spleen is a crucial secondary lymphoid organ that regulates the immune response between innate [macrophages, dendritic cells (DCs), natural killer (NK) cells, neutrophils, and monocytes] and adaptive (T and B cells) immune cells [6]. During immune cell activation, different cytokines [interferon (IFN)-γ, TNF-α, IL-4, IL-6, and IL-12] are secreted and other immune cells are sequentially stimulated, ultimately increasing the innate and adaptive immune cell population [6]. The intestine, another essential part of the immune system, correlates with immune cells in the intestinal lamina propria; these cells are regulated via pattern recognition receptors and are protected from physical [tight junctions; zonula occludens (ZO)-1, occludin, and claudin-1] and chemical [mucin-2 (MUC2)] barriers [7]. These physical barriers interact with MUC2, which is a glycosylated layer that covers epithelial cells to defend against immune imbalance-induced intestinal damage and disorders [8,9].

Medicinal herbs are well-known immunomodulators that have been used as ancient medicinal therapeutics. Notably, these herbs have lower drug resistance and fewer side effects than chemical drugs [10]. The development of herbal therapies has resulted in more effective treatments owing to the combination of various natural medicinal components that exhibit multiple biological effects [11]. *Angelica gigas*, one of the Umbelliferae family, is distributed throughout North Asia and has been used as a traditional remedy owing to its anticancer activity, which occurs via an immune stimulation mechanism involving the NF-κB and MAPK signals [12,13]. In East Asia, the root of the legume *Pueraria lobata* (Willd.) Ohwi (Fabaceae) is commonly used as a pharmacological resource because of its ability to stimulate immune-enhancing and hepatoprotective effects by inducing spleen activity [14,15]. *P. lobata* is rich in nutrients and antioxidant substances; thus, this herb is an excellent natural product for ameliorating stress damage [14]. Thus, we combined the water extracts of *A. gigas* roots and *P. lobata* flowers (CHL) and investigated the molecular mechanisms focusing on its immunostimulatory properties in RAW 264.7 cells and cyclophosphamide (CTX)-induced immunosuppression mice.

## 2. Results

### 2.1. CHL Increases the Immune Response in RAW 264.7 Macrophages

**Figure 1:**
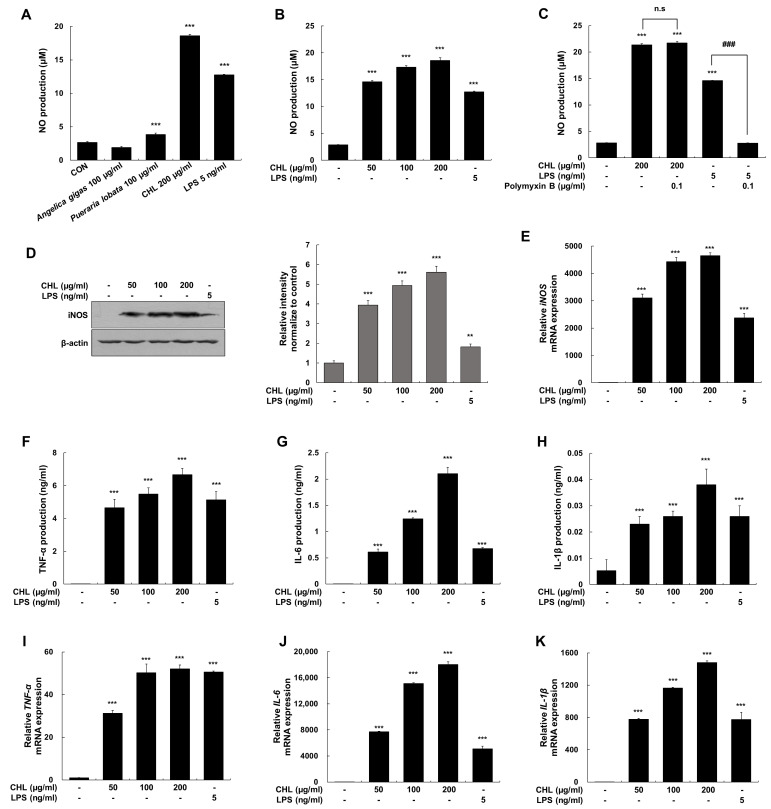
Effects of CHL on immune mediator production and expression in RAW 264.7 macrophages. (**A**,**B**) Cells were stimulated with *A. gigas* (100 μg/mL), *P. lobata* (100 μg/mL), CHL (50, 100, or 200 μg/mL), or LPS (5 ng/mL) for 24 h. (**C**) Cells were pretreated with polymyxin B (0.1 μg/mL) and then stimulated with CHL (200 μg/mL) or LPS (5 ng/mL) for 24 h. (**D**–**K**) Cells were stimulated with CHL (50, 100, or 200 μg/mL) or LPS (5 ng/mL). LPS was used as a positive control and β-actin was used as an internal control. Data are presented as mean ± SEM of three independent experiments. ** *p* < 0.01, *** *p* < 0.001 vs. CON; ### *p* < 0.001 vs. LPS-treated cells.

Assessment of the immune effect of CHL revealed that it potently increased NO production (18.61 ± 0.48 μM) in RAW 264.7 macrophages compared with *A. gigas* alone and *P. lobata* alone (1.98 ± 0.23 μM and 3.85 ± 0.21 μM, respectively; Figure 1A and Supplementary Figure S1A). NO production was enhanced by CHL; however, this increase was not affected by polymyxin B; a potent LPS-induced TLR4 antagonist. Therefore, the CHL-induced increase in NO is attributed to immune response rather than endotoxin contamination or non-specific cytotoxicity (Figure 1B,C, Supplementary Figure S1B,C). The protein and mRNA levels of iNOS were upregulated by CHL (Figure 1D,E). These findings, in conjunction with the results of TNF-α, IL-6, and IL-1β production and mRNA expression (Figure 1F–K), indicate that CHL-enhanced immune mediators may be related to macrophage activation.

### 2.2. CHL Stimulates the TLR2 Signaling Pathway in RAW 264.7 Macrophages

**Figure 2:**
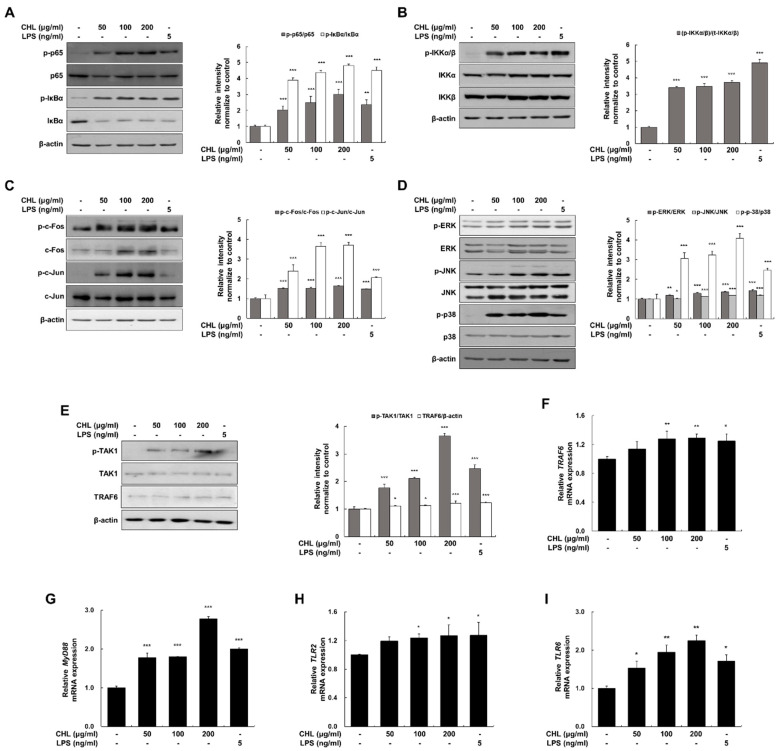
Effects of CHL on the TLR2/6 signaling pathway in RAW 264.7 macrophages. (**A**–**I**) Cells were stimulated with CHL (50, 100, or 200 μg/mL) or LPS (5 ng/mL) for 15–30 min or 6 h. LPS was used as a positive control and β-actin was used as an internal control. Data are presented as mean ± SEM of three independent experiments. * *p* < 0.05, ** *p* < 0.01, *** *p* < 0.001 vs. CON.

**Figure 3:**
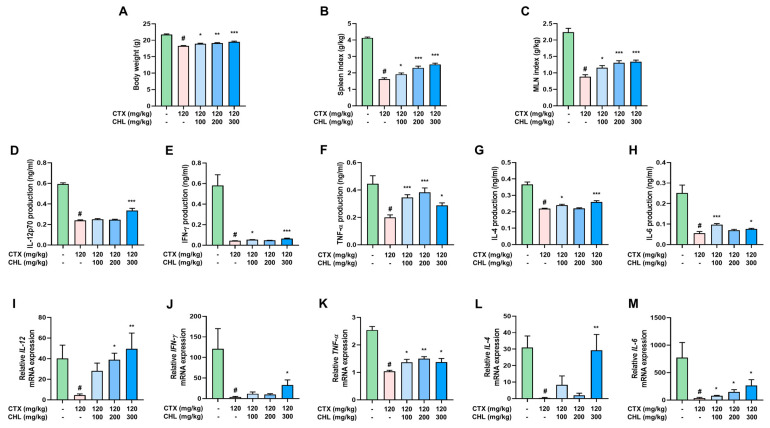
Effects of CHL on body weight, immune organ indices, and cytokine expression in CTX-treated mice. (**A**) Body weights and indices of (**B**) spleen and (**C**) MLN were measured at the end of the animal experiments. Data are presented as mean ± SEM (*n* = 23–24). (**D**–**H**) Cytokine production and (**G**–**M**) mRNA expression of IL-12, IFN-γ, TNF-α, IL-4, and IL-6. Data are presented as mean ± SEM (n = 7). # *p* < 0.05 vs. CON group; * *p* < 0.05, ** *p* < 0.01, *** *p* < 0.001 vs. CTX group.

As shown in Figure 2A,B, CHL upregulated the phosphorylation of p65 (NF-κB subunit), IκBα, and IKKα/β, and promoted the degradation of IκBα in RAW 264.7 macrophages. Consistently, CHL improved the phosphorylation of c-Fos and c-Jun (AP-1 subunits), and MAPK (ERK, JNK, and p38) (Figure 3C,D); stimulated the phosphorylation of TAK1 and TRAF6 (Figure 2E); and elevated the mRNA expression of *TRAF6*, *MyD88*, *TLR2*, and *TLR6* (Figure 2F–I) in RAW 264.7 macrophages by serving as a signal upstream of NF-κB and AP-1/MAPK. Complementarily, treatment with anti-TLR2 antibody reduced CHL- or peptidoglycan (PGN; TLR2 ligand)-induced NO production without inducing cytotoxicity (Supplementary Figure S1D,E). These findings suggest that the CHL regulates the TLR2-mediated immunostimulatory responses in RAW 264.7 macrophages.

### 2.3. CHL Preserves Body Weight, Immune Organ Index, and Cytokine Production in Immunosuppressed Mice

CHL was administered to an immunosuppressed animal model to validate the immune-enhancing effects observed in vitro. CTX (120 mg/kg, i.p.) reduced the body weight and indices of the spleen and mesenteric lymph node (MLN). However, these CTX-induced physiological changes were recovered by CHL (100, 200, or 300 mg/kg, p.o.) in immunosuppressed mice (Figure 3A–C). CHL regulated the production of IL-12, IFN-γ, TNF-α, IL-4, and IL-6 in the splenocytes of immunosuppressed mice. As shown in Figure 3D–H, except for TNF-α production, which was found to significantly recover following treatment with all the tested concentrations of CHL, the production of IL-12, IFN-γ, IL-4, and IL-6 was only mildly improved with 100 or 300 mg/kg CHL. Nonetheless, CHL increased concentration-dependent mRNA expression of IL-12 and IL-6 (Figure 3I,M). The mRNA expression levels of IFN-γ, TNF-α, and IL-4 followed the same pattern as that observed for cytokine production (Figure 3I–M). Notably, CHL did not affect blood GOT, GPT, BUN, and creatinine levels, suggesting no liver and kidney toxicity in mice with suppressed immune systems (Supplementary Figure S2A–D). Overall, CHL has immunomodulatory potential and can restore immune organ indices and cytokine levels in CTX-induced immunosuppressed mice.

### 2.4. CHL Recovers NK Cell Activity and Immune Cell Composition in Immunosuppressed Mice

**Figure 4:**
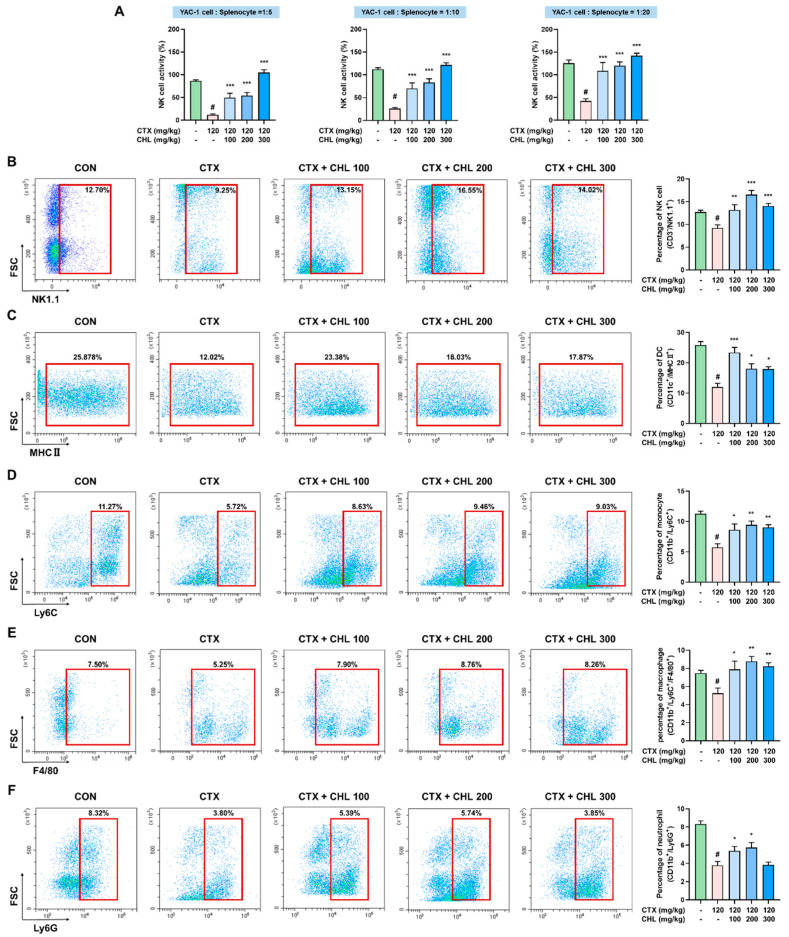
Effects of CHL on natural killer (NK) cell activity and characterization of innate immune cell population in CTX-treated mice. (**A**) NK cell activity of CHL, cell ratio between splenocytes and YAC-1 = 1:5, 1:10, or 1:20. Population of (**B**) CD3^−^/NK1.1^+^ NK cells, (**C**) CD11b^+^/MHC II^+^ dendritic cells, (**D**) CD11b^+^/Ly6C^+^ monocytes, (**E**) CD11b^+^/Ly6C^+^/F4/80^+^ macrophages, and (**F**) CD11b^+^/Ly6G^+^ neutrophils. Data are presented as mean ± SEM (*n* = 6–7). # *p* < 0.05 vs. CON group; * *p* < 0.05, ** *p* < 0.01, *** *p* < 0.001 vs. CTX group.

**Figure 5:**
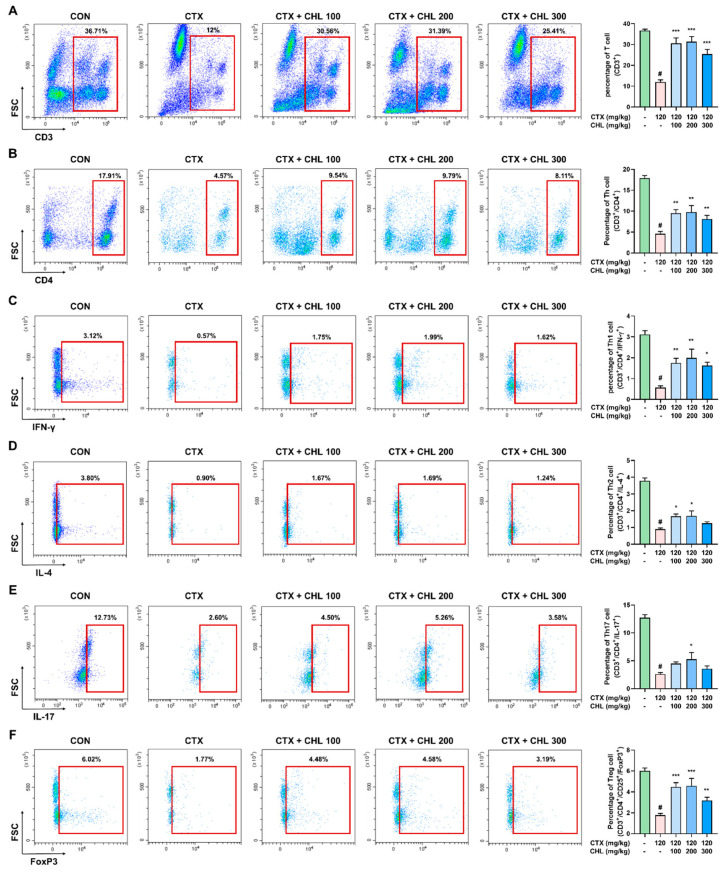
Effects of CHL on the characterization of the adaptive immune cell population in CTX-treated mice. Populations of (**A**) CD3^+^ T cells, (**B**) CD3^+^/CD4^+^ T helper cells, (**C**) CD3^+^/CD4^+^/IFN-γ^+^ Th1 cells, (**D**) CD3^+^/CD4^+^/IL-4^+^ Th2 cells, (**E**) CD3^+^/CD4^+^/IL-17^+^ Th17 cells, and (**F**) CD3^+^/CD4^+^/CD25^+^/FoxP3^+^ regulatory T cells. Data are presented as mean ± SEM (*n* = 6–7). # *p* < 0.05 vs. CON group; * *p* < 0.05, ** *p* < 0.01, *** *p* < 0.001 vs. CTX group.

The activity and population of immune cells were monitored to identify the immune cells that influence cytokine expression. NK cell cytotoxicity, a key indicator of immune response, was found to decrease in the CTX group compared to the CON group. At ratios of 1:5, 1:10, and 1:20; the percentage of NK cell cytotoxicity: 11.99% ± 0.61%, 25.95% ± 0.78%, and 42.12% ± 1.60%, respectively, were obtained in the CTX group. In contrast, higher NK cell cytotoxicity of 86.44% ± 0.76%, 112.45% ± 0.96%, and 125.99% ± 2.13% were obtained in the CON group at the same ratios. CHL significantly restored the reduced NK cell activity in the CTX-induced immunosuppressed mice (CHL 100: ratio of 1:5, 1:10, or 1:20 = 49.53% ± 3.25%, 69.87% ± 4.11%, or 108.37% ± 5.87%, respectively; CHL 200: ratio of 1:5, 1:10, or 1:20 = 54.19% ± 2.22%, 82.86% ± 2.86%, or 120.10% ± 2.64%, respectively; CHL 300: ratio of 1:5, 1:10, or 1:20 = 104.59% ± 2.05%, 121.50% ± 1.66%, or 141.90% ± 1.86%, respectively) (Figure 4A). The populations of innate immune cells (NK cells, DCs, monocytes, macrophages, and neutrophils; Figure 4B–F), and adaptive immune cells (T, Th, Th1, Th2, Treg, Th17, Tc, and B cells; Figure 5A–F, Supplementary Figure S3A,B) were potently decreased in the CTX group, but treatment with CHL increased the number of immune cells. Although CHL did not induce dose-dependent recovery of these immune cells in CTX-induced immunosuppressed mice, oral administration of CHL (100 and 200 mg/kg) restored the levels of most innate and adaptive immune cells. However, even at the high dose of CHL, no significant recovery of neutrophils, Th2 cells, and Th17 cells was achieved. These results indicate that NK cell activity, some immune cell population, and cytokine production may be involved in the immune-enhancing activity of CHL in CTX-induced immunosuppressed mice.

### 2.5. CHL Protects the Intestinal Tissue by Regulating the Tight Junctions and Mucin 2 in Immunosuppressed Mice

**Figure 6:**
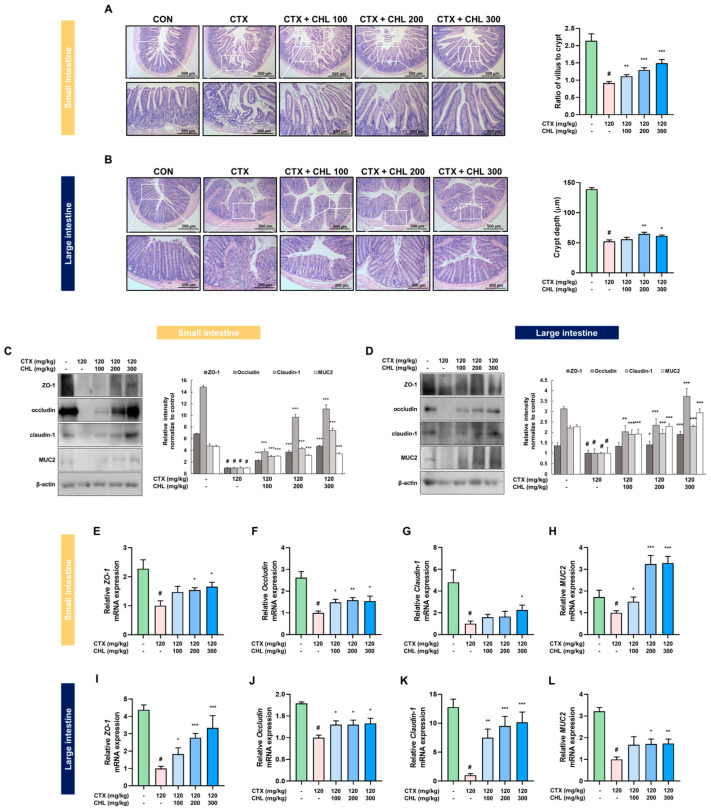
Effects of CHL on histological changes in the intestine and the expression of tight junction markers and mucin 2 in CTX-treated mice. Histological changes in the (**A**) small intestine and (**B**) colon. (**C**–**L**) Protein and mRNA expression of tight junction-related markers (ZO-1, occludin, and claudin-1) and MUC2 in the small and large intestines. Data are presented as mean ± SEM (*n* = 6–7). # *p* < 0.05 vs. CON group; * *p* < 0.05, ** *p* < 0.01, *** *p* < 0.001 vs. CTX group.

Compared to the CTX group, histological changes and a modulated ratio of the villus to the crypt (Figure 6A) and crypt depth (Figure 6B) were observed in the intestines of CHL groups. Accordingly, the protein and mRNA expressions of ZO-1, occludin, claudin-1, and MUC2 were downregulated in the CTX group. Notably, CHL prevented these reductions (Figure 6C–L). Taken together, these findings suggest that CHL may induce immune cell recovery by upregulating protective mediators in the intestines of CTX-induced immunosuppressed mice.

### 2.6. CHL Regulates Gut Microbiome Composition in Immunosuppressed Mice

**Figure 7:**
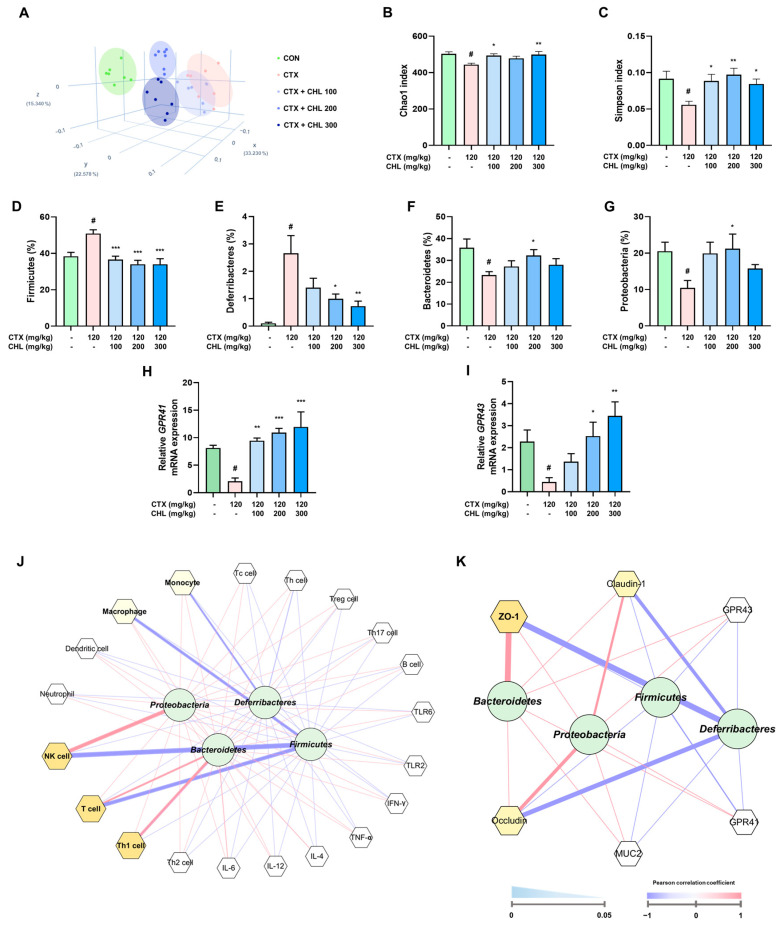
Effects of CHL on gut microbiome composition in CTX-treated mice. Analysis of microbial diversity: (**A**) Principal coordinate analysis (PCoA) plots; (**B**) Chao1 index; (**C**) Simpson index between each group. (**D**–**G**) The relative ratio of *Firmicutes*, *Deferribacteres*, *Bacteroidetes*, and *Proteobacteria.* (**H**,**I**) *GPR41* and *GPR43* mRNA expression in the colon. Correlation analysis between gut microbiota and (**J**) immune response and (**K**) intestinal immunity. Data are presented as mean ± SEM (*n* = 6–7). # *p* < 0.05 vs. CON group; * *p* < 0.05, ** *p* < 0.01, *** *p* < 0.001 vs. CTX group.

Based on the principal coordinate analysis plot, the CTX shifted away from the CON group; however, this shift was attenuated by CHL (Figure 7A). Notably, based on the Chao1 and Simpson indices, CHL restored the α-diversity in CTX-induced immunosuppressed mice (Figure 7B,C). Furthermore, in the β-diversity analysis, CTX markedly increased the *Firmicutes* ratio to *Deferribacteres* and decreased the ratio of *Bacteroidetes* to *Proteobacteria*; however, CHL considerably ameliorated gut dysbiosis (Supplementary Figures S4 and S7D–G). Furthermore, receptors related to metabolites of microorganisms in the intestine were evaluated to supplement the effect of CHL on the restoration of dysbiosis. CHL restored the mRNA expression of receptors, such as *GPR41* and *GPR43*, in CTX-induced immunosuppressed mice (Figure 7H,I). As a result, a functional correlation analysis was performed between changes in the CHL-mediated gut microbiome, immune response, and intestinal metabolites to identify the immune response mediators that were preferentially targeted by CHL for effective immune enhancement. As shown in Figure 7J, *Firmicutes* and *Deferribacteres* were negatively associated with NK cells, T cells, macrophages, and monocytes; however, *Bacteroidetes* and *Proteobacteria* were positively correlated with NK cells, T cells, and Th1 cells. Although the associations with *Firmicutes* and *Deferribacteres* were negative, *Bacteroidetes* and *Proteobacteria* were positively associated with ZO-1, occludin, and claudin-1 (Figure 7K). Taken together, CHL may play a role in modulating the symbiotic relationship of the gut microbiome and provide the major metabolites required to regulate immune responses in CTX-induced immunosuppressed mice.

### 2.7. CHL Modulates Intestinal Immunity via TLR2/6 Signaling Pathway in Immunosuppressed Mice

**Figure 8:**
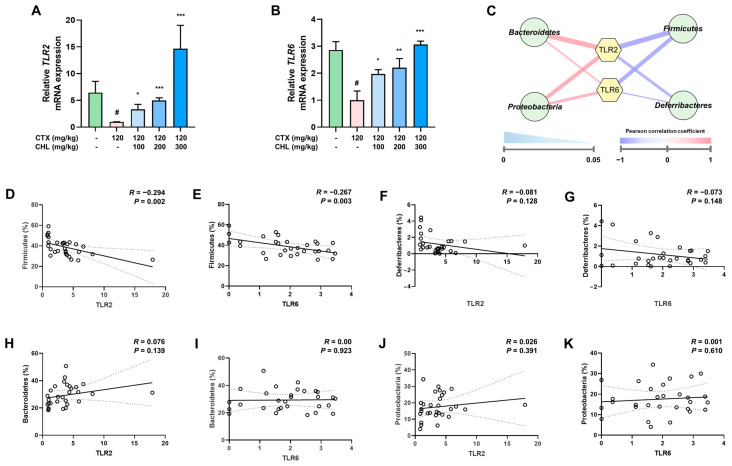
Effects of CHL on TLR2/6 signaling pathway in CTX-treated mice. (**A**) TLR2 and (**B**) TLR6 mRNA expressions of the small intestine. Data are presented as the means ± SEM (*n* = 7). # *p* < 0.05 vs. CON group; * *p* < 0.05, ** *p* < 0.01, *** *p* < 0.001 vs. CTX group. Correlation analysis between gut microbiota and (**C**) TLR2/6. Regression analysis between TLR2/6 and (**D**,**E**) Firmicutes; (**F**,**G**) Deferribacteres; (**H**,**I**) Bacteroidetes; and (**J**,**K**) Proteobacteria. R means correlation coefficient value and P means *p* value.

To determine whether CHL, which induced RAW 264.7 macrophage activation via TLR2/6 signaling in vitro studies, also activated the TLR2/6 pathway in vivo, experiments were conducted using intestinal tissues. As shown in Figure 8A,B, we found that the mRNA expression levels of TLR2 and TLR6 were downregulated in the CTX group. However, these levels were restored in the CHL treatment group. Correlation analysis with gut microbiota, which plays a crucial role in regulating the immune system, revealed a positive correlation between TLR2/6 and Bacteroidetes and Proteobacteria, while a negative correlation was observed with Firmicutes and Deferribacteres (Figure 8C). Furthermore, regression analysis was conducted for quantitative validation, the regression analysis showed that there was only a significant association between Firmicutes and TLR2 (R = −0.294; *p* = 0.002) and TLR6 (R = −0.267; *p* = 0.003) at the phylum level (Figure 8D–K). These findings suggest that CHL regulated intestinal immunity through the TLR2/6 pathway and that this regulation is particularly associated with Firmicutes in CTX-induced immunosuppressed mice.

## 3. Discussion

Medicinal herbs and their extracts are known as safe therapeutic agents that possess immunomodulatory properties. These natural remedies are often combined to treat illnesses, such as psychosomatic, nervous system, and inflammatory disorders [16]. Among these, *A. gigas* and *P. lobata* are known modulators of immune responses [12,14]. Notably, *A. gigas*, one of the main components of “HemoHIM^®^”, which is recognized as an immune response regulator in Korea, has demonstrated potential as an immune mediator; however, mechanistic studies on its action are currently limited [17,18]. In the case of *P. lobata*, only in vitro experiments, such as confirmation of MAPK signaling, were conducted for research on immune-enhanced effects [19], but additional research is needed to confirm its efficacy in immune regulation. We hypothesized that the combination of *A. gigas* and *P. lobata* would yield outstanding immunostimulatory effects. Based on the aforementioned studies, different proportions of *A. gigas* and *P. lobata* were combined. Based on our screening process, NO production was significantly enhanced at a 5:5 ratio, with no observable cytotoxicity (Supplementary Figure S7A,B). We then examined the immune-enhancing effects of CHL and elucidated its detailed mechanisms in vitro and in vivo. Macrophages are representative innate immune cells that provide energy to support the activation of the immune system and directly affect adaptive immunity by controlling transcription factors [20]. Activated macrophages serve as the representative marker of the immune response by producing NO and immunostimulatory cytokines, TNF-α, IL-6, and IL-1β, via NF-κB and MAPK activation. The upstream signaling pathway of transcription factors is activated via the interaction of TAK1 with TRAF6, leading to the phosphorylation of NF-κB and AP-1 signaling pathways through IκBα/IKKα/β or MAPK activation, respectively [21,22]. The MyD88-dependent TLR 2 pathway, which is crucial for regulating both innate and adaptive immune cells in the immune response, is influenced by activated TAK1 and TRAF6 signaling cascade [23]. TLR6 is the co-receptor that works alongside TLR2 in mediating immune responses, only a few studies have been conducted on the immune-stimulatory effects of this TLR2/6 combination [24]. Consistent with the findings of previous studies, it was demonstrated that CHL facilitates an immune response by inducing the secretion of immune mediators and activating the TLR2/6 signaling pathways in RAW 264.7 cells. Although this study focused on TLR2 as an initial upstream mechanism and used an anti-TLR2 antibody, we were unable to confirm the role of TLR6 in the CHL immunostimulatory mechanism as part of the TLR2/6 heterodimer. It is clear that CHL induced immune enhancement in RAW 264.7 cells without cell damage, but additional research on the interaction between CHL and TLR2/6 is required.

To verify the immunostimulatory effects of CHL in vitro, we proceeded to determine its effects on CTX-treated immunosuppressed mice. High doses of CTX (up to 120 mg/kg) function as an alkylating agent, causing DNA damage-induced cytotoxicity and severe immunosuppression [25]. According to these characteristics, CTX decreases body weight and the immune organ index, which serve as representative biomarkers of immune system responses [26]. The spleen plays a crucial role in regulating whole immunity and activating T and B cells [6]. The MLN, located in the peritoneal area, is one of the lymph nodes that produces immune responses by interacting with intestinal immune cells against immune imbalance [27]. Herein, CHL was found to restore the decreased body weight and spleen and MLN indices in CTX-induced immunosuppressed mice. Activated splenocytes maintain immune balance by promoting the interaction of Th1 cells, Th2 cells, and macrophages, as well as cytokine secretion [6,28]. Th1 and Th2 cells, as the major regulator of adaptive immune responses, secrete immunostimulatory cytokines (Th1 cells: IFN-γ and TNF-α; Th2 cells: IL-4 and IL-6) [29]. In addition, macrophages (IL-12), one of the major innate immune cells, interact with T cells to maintain homeostasis against the abnormal immune system [28]. High doses of CTX have been found to induce an imbalance in the immune system by reducing the generation of IL-12, IFN-γ, TNF-α, IL-4, and IL-6 in CTX-treated mice models [30,31]. Based on our findings, CHL enhanced general cytokine production and mRNA expression in CTX-induced immunosuppressed mice; however, the expressions of IFN-γ and IL-4 were not recovered by CHL compared to those of other cytokines. We predicted that these changes would contribute to immune responses primarily driven by the host’s tendency to maintain homeostasis [32]. As various immune cells become activated, the system inherently regulates itself to prevent excessive immune responses. Therefore, it is expected that in CHL-treated CTX-induced immunosuppressed mice, certain cells may not have been fully activated due to this homeostatic regulation. However, the balance in Th1/Th2 cells, which plays a key role in regulating immune imbalance therapies [33], must be examined in future studies. NK cells are lymphocytes of the innate immune system in the spleen that exhibit cytotoxic activities and spontaneously generate systemic immune responses to counteract abnormal immune function [34]. As NK cell activity was used as a representative indicator of immune response activation, CTX was previously found to reduce NK cell activity [35]. However, the NK cells’ cytotoxicity in the CTX group was increased by CHL. Therefore, this upregulated activity may affect the population of NK and immune cells in CTX-induced immunosuppressed mice. Indeed, treatment with CTX was found to decrease both innate (including NK cells, DCs, neutrophils, monocytes, and macrophages) and adaptive (containing T, Th, Th1, Th2, Tc, Treg, Th17, and B cells) immune cell populations. In contrast, treatment with CHL ameliorated the abnormal immune cell populations induced by CTX. As the concentration-dependent relationship between CHL and recovery of immune cell populations could not be confirmed, further studies are needed to determine the optimal CHL concentration.

The intestine, the largest component of the immune system, is divided into two parts according to the physiological characteristics of the small intestine and the colon [7]. The small intestine plays a crucial role in the absorption of metabolites owing to its villi, in contrast, the colon harbors numerous commensal microorganisms that are key to the maintenance of gut integrity and homeostasis [7,36]. The intestine is protected by the intestinal mucosa, which chemically defends the surface of the intestinal tract, and is produced by goblet cells in the crypt. Tight junctions provide a physical barrier using conjugate junctional components. Innate and adaptive immune cells are located in the lamina propria in response to immune imbalance [7,37]. Some studies have found structural destruction and decreased expression of tight junction markers in the small intestine and colon of CTX-treated mice [30,38]. In the present study, we found histological damage, lower villus-to-crypt ratio, and reduced protein and mRNA expression of tight junctions and mucus in the intestine of CTX-induced immunosuppressed mice. Contrary to treatment with CTX, CHL restored these disproportions to maintain intestinal homeostasis. Furthermore, during the immune imbalance, pattern recognition receptors, especially TLR2 (a highly expressed receptor in the colon), regulate TLR2 in parallel with downstream signals in CTX-induced immunosuppressed mice [39]. Although TLR6 is not the main receptor in the colon, it regulates the intestinal immune system by modulating the immune response to sense changes in host immunity [40]. Consistent with previous studies, our data showed that the mRNA expression levels of *TLR2* and *TLR6* were restored in the CHL treatment group. Therefore, we suggested that CHL may help prevent intestinal leakage and potentially enhance intestinal immunity by strengthening tight junction markers.

The gut microbiome is a major immune-regulatory mediator. As a result, studies have been performed to develop modulators, including herbs, that can regulate intestinal immune function [41]. Previously, the administration of an herbal complex restored normal levels of α- and β-diversity and short-chain fatty acids (SCFAs) in gut dysbiosis [42]. Similarly, our findings showed that CHL administration ameliorated CTX-induced gut dysbiosis in terms of both α- and β-diversity. Although SCFA analysis could not be performed, we quantified the mRNA expression of G-protein coupled receptor (GPR) in CTX-induced immunosuppressed mice. GPR, located in intestinal epithelial cells, is activated by SCFA produced by gut bacteria and induces an immune response via TLR interaction [43]. Notably, the mRNA expression levels of *GPR41* and *GPR43* were decreased in CTX-induced immunosuppressed mice; however, these reductions were reversed by treatment with CHL. A correlation analysis was further performed to identify the key factors involved in CHL-mediated interactions between the gut microbiome and the immune system. In the CHL and CTX co-treated immunosuppression model, *Bacteroidetes* and *Proteobacteria* were found to have a strong positive correlation with NK cells, Th1 cells, and ZO-1. While these results, in *Firmicutes* and *Deferribacteres*, a negative correlation was observed with the immune markers (NK cells, Th1 cells, and ZO-1). According to these, gut bacteria interact with TLRs to mediate immune regulation. A correlation and regression analysis between TLR2/6 and gut microbiota showed that there was only a significant association between *Firmicutes* and TLR2/6 at the phylum level. These findings are consistent with other studies, suggesting that *Bacteroidetes* are associated with lymphocyte activation and tight junctions in abnormal gut microbiomes [44]. However, research on the interaction between *Firmicutes* and TLRs remains limited, underscoring the need for a knockout mouse model to elucidate the activation mechanism of CHL in a CTX-induced immunosuppressed model. Nevertheless, given the clear evidence of CHL’s efficacy in enhancing immunity, these findings collectively support the potential for developing immune modulators with improved effects. Moreover, CHL, with its remarkable efficacy, is expected to contribute not only to the development of healthy functional foods with reduced side effects compared to current therapeutics but also to a broader application as a vaccine adjuvant. However, despite the remarkable immunostimulatory effects of CHL, its potential interactions with immunosuppressive agents must be carefully considered. The concurrent use of CHL may influence immune responses, potentially reducing the efficacy of immunosuppressive therapy or leading to unintended immune activation. Therefore, further studies are warranted to assess the safety and compatibility of CHL in individuals undergoing immunosuppressive treatment.

## 4. Materials and Methods

### 4.1. Preparation of A. gigas and P. lobata Extracts

The washed and dried A. gigas roots and P. lobata flowers were extracted twice with distilled water (95 °C, 4 h). Each extract was then concentrated and dried. Sequentially, those extracts were combined at a 1:1 ratio, and this mixed standardized powdered extract (CHL) was provided by CH Labs (Seoul, Republic of Korea). All purchase directions have been added to Supplementary Table S1.

### 4.2. High-Performance Liquid Chromatography (HPLC) Analysis of Nodakenin and Tectoridin in CHL

**Figure 9:**
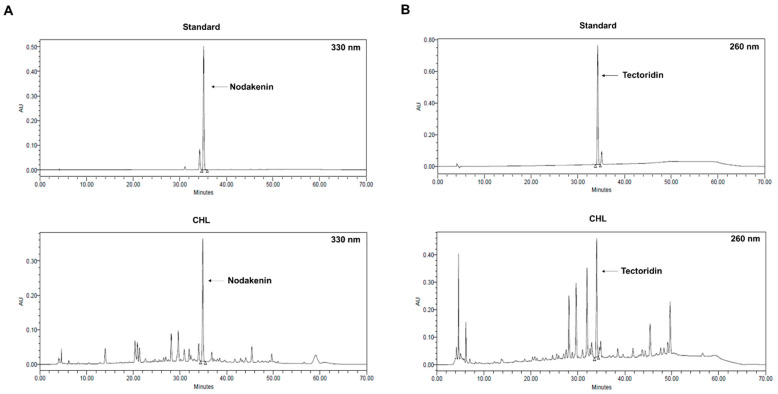
Representative HPLC chromatograms of CHL and standards (nodakenin and tectoridin). HPLC chromatograms of (**A**) nodakenin standard and CHL at 330 nm and (**B**) tectoridin standard and CHL at 260 nm.

HPLC and chromatographic separations (YMC Triart C18, 250 × 4.6 mm, 5 μm) were used to analyze the CHL. The mobile phases were distilled water containing 10% (*v*/*v*) formic acid (solvent A) and methanol: acetonitrile: distilled water: formic acid (22.5:22.5:40:10, *v*/*v*/*v*/*v*) (solvent B). The elution profile was 0-5 min, 5% B; 5–40 min, 5–75% B; 40–45 min, 75–100% B; 45–55 min, 100% B; 55–60 min, 100–5% B; 60–70 min, 5% B at flow rate 0.7 mL/min (injection volume: 10 μL). UV detection of nodakenin and tectoridin was performed at 330 and 260 nm. The results of the HPLC analysis of CHL and its active compounds are shown in Figure 9. All purchase directions have been added to Supplementary Table S1.

### 4.3. Cell Culture and Sample Treatment

RAW 264.7 cells were incubated in Dulbecco’s modified Eagle’s medium [(with fetal bovine serum (10%) and penicillin and streptomycin (1%)] in a 37 °C, 5% CO_2_ atmosphere. RAW 264.7 cells were treated with CHL (50, 100, or 200 μg/mL) or lipopolysaccharide (LPS) (5 ng/mL) at various times.

### 4.4. Nitrite Assay

The nitrite content was measured as previously described [45]. RAW 264.7 cells were treated with CHL (50, 100, or 200 μg/mL) or LPS (5 ng/mL) for 24 h. Nitrite from the cells’ supernatants was determined using Griess reagent, and the absorbance was measured at 540 nm.

### 4.5. Real-Time Quantitative PCR (RT-qPCR)

According to previous research [46], RNA from intestine and lung tissues was obtained using Easy blue^®^ kits. cDNA was synthesized using 0.5 mg/mL random oligonucleotide primers and TOPscript^TM^ RT DryMIX. PCR was performed for 50 cycles under the following conditions: denaturation at 95 °C for 5 s, annealing at the respective Tm (°C) for 10 s, and elongation at 72 °C for 20 s. The levels of target gene expression were analyzed by measuring the incorporation of TB green using the QuantStudio^TM^ 1 System (Supplementary Table S2).

### 4.6. Western Blot Analysis

Proteins were extracted and detected as previously reported [46]. Proteins from intestinal tissues were obtained using the PRO-PREP™ protein extraction solution. Protein concentrations were determined using the Bradford reagent following the manufacturer’s instructions. The proteins were resolved on a 10–15% polyacrylamide gel and transferred onto a polyvinylidene difluoride (PVDF) membrane. The PVDF membrane was incubated in 1–5% skim milk with a primary antibody at 4 °C for 18 h. The immunoblotted membrane was washed three times with Tris-buffered saline/Tween 20 (TBS/T) and incubated with a secondary antibody at 25 °C for 2 h, then the proteins were detected using an ECL chemiluminescence substrate. Information on antibodies is indicated in Supplementary Table S3.

### 4.7. Determination of Cytokines Production

RAW 264.7 macrophages were treated with CHL (50, 100, or 200 μg/mL) or LPS (5 ng/mL) for 24 h. Isolated splenocytes were seeded at 3 × 10^6^/cells and treated with concanavalin A (5 μg/mL) for 48 h. The cytokines productions in the supernatant of RAW 264.7 macrophages and splenocytes were determined using mouse duoset ELISA kits at 450 nm absorbance.

### 4.8. Designing Animal Experiments Scheme

Adult male mice (C57BL/6) were maintained under steady external status (temperature: 20 ± 2 °C, humidity: 40–60%, light/dark cycle: 12 h). Animal experiments followed the guidelines of the Ethics Committee for Animal Care and Use of Kyung Hee University (KHSASP-23-273). Mice were grouped per (n = 10/group): (i) CON (vehicle + saline 200 μL), (ii) CTX (vehicle + CTX 120 mg/kg), (iii) CHL 100 + CTX (CHL 100 mg/kg/day + CTX 120 mg/kg), (iv) CHL 200 + CTX (CHL 200 mg/kg/day + CTX 120 mg/kg), and (v) CHL 300 + CTX (CHL 300 mg/kg/day + CTX 120 mg/kg). During the experiments, mice were orally administrated CHL (dissolved in deionized water, 200 μL) for 16 days, intraperitoneally injected with saline (200 μL) or CTX (dissolved in saline, 200 μL) on the 12th, 13th, and 14th days and sacrificed on the 18th day of the experiments (Supplementary Figure S6). All purchase directions have been added to Supplementary Table S1.

### 4.9. Splenocyte Isolation and Preparation

The isolated spleen was prepared with medium and then passed through a cell strainer. Splenocytes were centrifuged (1000× *g*, 25 °C, 10 min), and eliminated the red blood cells with eBioscience^TM^ 1 × RBC Lysis Buffer for 5 min and cells were centrifuged (1000× *g*, 25 °C, 3 min) and then were resuspended with RPMI medium.

### 4.10. Determination of NK Cell Activity

Cytotoxic activity [(effector cells to target cells) = (NK cells to YAC-1 cells)] was estimated through a lactate dehydrogenase (LDH) detection kit. Isolated splenocytes were seeded in the following ratios (YAC-1 cells: splenocytes = 1:5, 1:10, or 1:20), and mIL-2 (50 ng/mL), as a T cell activator, was added for 48 h. After that, target cells (4 × 10^5^ cells/well) were co-cultured for 4 h and supernatants were superinduced to the LDH substrate mixture. The NK cells’ activity was calculated by the following equation: cytotoxicity (%) = [(experimental LDH-spontaneous LDH)/(maximum LDH-spontaneous LDH)] × 100 at 490 nm.

### 4.11. Flow Cytometry

Isolated splenocytes (1 × 10^5^ cells) were stained with an anti-mouse CD16/32 antibody for 10 min and targeted fluorophore-coated antibodies in the dark (Supplementary Table S4) for 20 min. The marked cells were analyzed by CytoFLEX (Supplementary Figure S7).

### 4.12. Gut Microbiome and Correlation Analysis

Gut microbiome analysis proceeded as described previously [47]. Amplified DNA analysis was measured using the Illumina iSeq 100 Sequencing System. Correlation analysis was progressed by the software Cytoscape program (v3.10.1) and a visualized network was built by MetScape, using Pearson correlation coefficients.

### 4.13. Statistical Analysis

Each result is expressed as means ± standard error of the mean (SEM) for in vitro and in vivo. Statistical significance (*p* < 0.05) was analyzed by analysis of variance (ANOVA) and Dunnett’s post hoc test using GraphPad Prism 8.0.2.

## 5. Conclusions

In summary, our research demonstrated that CHL exerts immune-enhancing effects in RAW 264.7 macrophages by correlating with the TLR2/6-mediated NF-κB and MAPK signaling pathways. Additionally, in a CTX-induced immunosuppressed mouse model, CHL facilitated the recovery of systemic immunity in the spleen, a secondary lymphoid organ, restored intestinal immunity, and promoted stabilization of the gut microbial community. These immunoregulatory effects appear to be influenced by the interaction between TLR2/6 signaling and the intestinal microbiota. Taken together, these findings highlight CHL as a promising candidate for host immunity enhancement and has the potential for studying the immune response mechanisms of TLR2/6 heterodimers.

## Data Availability

The data analyzed in this study are available from the first or corresponding author upon reasonable request.

## Supplementary Materials

The following supporting information can be downloaded at: https://www.mdpi.com/article/10.3390/ph18030336/s1, Table S1: Purchase information on materials; Table S2: Primer sequences used in RT-qPCR; Table S3: Antibodies used in western blot analysis; Table S4: Antibodies used in flow cytometry; Figure S1: Effects of *Angelica gigas*, *Pueraria lobata*, and CHL on cell viability in RAW 264.7 macrophages; Figure S2: Effects of CHL on GOT, GPT, BUN, and creatinine production in cyclophosphamide (CTX)-induced mice.; Figure S3: Effects of CHL on cytotoxic T cell and B cell populations in CTX-treated mice; Figure S4: Effects of CHL on the relative ratio of gut microbiota composition (phylum) in CTX-induced mice (n = 7); Figure S5: Effects of combination ratio of *A. gigas* and *P. lobata* on cell viability and NO production in RAW 264.7 macrophages; Figure S6: Scheme of animal experiments. Establishment of CTX-induced immunosuppressed mice model and oral administration of CHL (100, 200, or 300 mg/kg/day) for 16 days; Figure S7: Gating methods of immune cells‘ population in CTX-treated mice.

## Abbreviations

ANOVA, analyzed by analysis of variance; AP-1, activator protein-1; CHL, water extract of *A. gigas* roots and *P. lobata* flowers; CTX, cyclophosphamide; DCs, dendritic cells; GPR, G-protein coupled receptor; HPLC, high-performance liquid chromatography; IL, interleukin; IκB, inhibitor of NF-κB; IFN, interferon; IKK α/β, IκB kinase α/β; iNOS, inducible NO synthase; LPS, lipopolysaccharide; LDH, lactate dehydrogenase; MUC2, mucin-2; MyD88, myeloid differentiation primary response 88; MLN, mesenteric lymph node; MAPK, mitogen-activated protein kinase; NF-κB, nuclear factor kappa B; NK, natural killer; NO, nitric oxide; PGN, peptidoglycan; RPMI, Roswell Park Memorial Institute; SEM, standard error of the mean; TNF-α, tumor necrosis factor-α; TAK1, transforming growth factor β-activated kinase 1; TRAF6, tumor necrosis factor receptor-associated factor 6; TLR, toll-like receptor; ZO, zonula occludens
