# Supplementary material for: Immunomodulatory Effects of a Standardized Botanical Mixture Comprising Angelica gigas Roots and Pueraria lobata Flowers Through the TLR2/6 Pathway in RAW 264.7 Macrophages and Cyclophosphamide-Induced Immunosuppression Mice
Source: Pharmaceuticals (Basel). 2025 Feb 27;18(3):336. doi: 10.3390/ph18030336 (PMC11944983; doi:10.3390/ph18030336)
Supplement: Supplementary file 1 [file pharmaceuticals-18-00336-s001.zip › pharmaceuticals-3480735-supplementary.pdf]

Supplementary methods

MTT assay

Cell viability was measured by previous research [1]. RAW 264.7 macrophages (2×10^5/well) were seeded in a 24-well plate and treated with CHL (50, 100, or 200 μg/mL) or LPS (5 ng/mL) for 24 h.

Biochemistry analysis in blood

To collect plasma, blood was centrifugated (845 × g, 25℃, 10 min) and was analyzed by three parts of toxic evaluation; glutamate oxaloacetate transaminase (GOT), glutamate pyruvate transaminase (GPT), blood urea nitrogen (BUN), and creatinine levels, through T&P Bio (Gwangju, Korea).

Histopathology analysis

The small and large intestinal tissues were fixed in 4% paraformaldehyde for 18 h. The intestinal tissues were divided on the slides for assessment of tight junction stability, and part of the intestinal tissues were stained with H&E solution.

Supplementary tables

Supplementary Table S1. Purchase information on materials.

Purchase	Materials
Bolak Co. Ltd.<br>(Hwaseong, *Republic of* Korea)	*A. gigas* roots and *P. lobata flowers*
Korean Cell Line Bank<br>(Seoul, *Republic of* Korea)	RAW 264.7 macrophages
Intron Biotechnology<br>(Seoul, Korea)	Easy blue® kits, PRO-PREP™ protein extraction solution
Thermo Fisher Scientific<br>(Waltham, MA, USA)	QuantStudioTM1 Real-Time PCR Instrument
TaKaRa<br>(Shiga, Japan)	TB Green® Premix Ex Taq™ (Tli RNaseH Plus)
Enzynomics<br>(Daejeon, *Republic of* Korea)	TOPscript™ RT DryMIX
Promega<br>(Madison, WI, USA)	Random oligonucleotide primers
Bio-Rad Laboratories<br>(Hercules, CA, USA)	Bradford reagent
Santa Cruz Biotechnology<br>(Dalla, TX, USA)	ECL chemiluminescence substrate
YMC<br>(Sungnam, *Republic of* Korea)	YMC Triart C18
Thermo Fisher Scientific<br>(Waltham, MA, USA)	QuantStudio™1 Real-Time PCR Instrument
R&D Systems<br>(NE Minneapolis, MN, USA)	Mouse duoset ELISA kits
Invitrogen<br>(Carlsbad, CA, USA)	eBioscience™ 1 × RBC Lysis Buffer
Sigma-Aldrich<br>(St. Louis, MO, USA)	Nodakenin, tectoridin, polymyxin B sulfate, lipopolysaccharide (LPS) from *Escherichia coli*, cyclophosphamide (CTX), lactate dehydrogenase (LDH) cytotoxicity detection kit, and chemicals
Invivogen<br>(San Diego, CA, USA)	Anti-mTLR2-IgG and Mouse Control IgG2a antibodies

Hyclone (Logan, UT, USA)	Dulbecco's modified Eagle's medium (DMEM), Roswell Park Memorial Institute medium (RPMI), fetal bovine serum (FBS), and penicillin and streptomycin (PS)
Waters (Milford, MA, USA)	E2695 Separation Module HPLCs and photodiode array detector
Orient Bio Inc (Seongnam-si, *Republic of* Korea)	Adult male C57BL/6 mice
BioLegend Inc. (Sandiego, CA, USA)	Fluorophore-coated antibodies
Beckman Coulter (Kraemer Boulevard, CA, USA)	CytoFLEX
Illumina (San Diego, CA, USA)	Illumina iSeq 100 Sequencing System
GraphPad Software Inc. (San Diego, CA, USA)	GraphPad Prism 8.0.2.

Supplementary Table S2. Primer sequences used in RT-qPCR.

Gene	Forward sequence	Reverse sequence	Tm (℃)
Claudin-1	ACAGTGCAAAGTCTTCGATT	ATCTTCTGCACCTCATCATC	54
GPR41	CGACTAGAGATGGCTGTGGT	AGAAGATGAGCAGTGTGGCT	58
GPR43	TCCAGCCTGGCTTTCCAATA	GCCTGCAGGAGACATTTCAG	58
Occludin	TGGCGGATATACAGACCCAA	CGATCGTGGCAATAAACACC	57
IFN-γ	GCTGATCCTTTGGACCCTCT	AGAGCTGCAAAGCCAAGATG	58
IL-4	TTGTCATCCTGCTCTTCTTT	TCTTCTTCAAGCATGGAGTT	54
IL-6	GAGGATACCACTCCCAACAGACC	AAGTGCATCATCGTTGTTCATACA	59
IL-12	TCTGCAGAGAAGGTCACACT	ATGAAGAAGCTGGTGCTGTA	57
IL-1β	ACCTGCTGGTGTGTGACGTT	TCGTTGCTTGGTTCTCCTTG	55
iNOS	AATGGCAACATCAGGTCGGCCATCACT	GCTGTGTGTCACAGAAGTCTCGAACTC	55
MUC2	ATGCCCACCTCCTCAAAGAC	GTAGTTTCCGTTGGAACAGTGAA	58
MyD88	CCCCACTCGCAGTTTGTTGG	CTGGCAGTCCTCCTCGATGC	60

TLR2	GTTGTTCCCTGTGTTGCTGG	GAGTTCGCAGGACCAAACAA	58
TNF-α	AGCACAGAAAGCATGATCCG	CTGATGAGAGGGAGGCCATT	56
TRAF6	CATCTTCAGTTACCGACAGCTCAG	TGGTCGAGAATTGTAAGGCGTAT	59
ZO-1	CTTCTCTTGCTGGCCCTAAAC	TGGCTTCACTTGAGGTTTCTG	58
β-actin	ATCACTATTGGCAACGAGCG	TCAGCAATGCCTGGGTACAT	58

Supplementary Table S3. Antibodies used in Western blot analysis.

Purchase	Antibody
Santa Cruz Biotechnology Inc. (Dallas, Texas, USA)	iNOS (sc-650), p65 (sc-8008), IκBα (sc-371), c-Fos (sc-253), p-c-Jun (sc-822), p-ERK (sc-7383), ERK (sc-94), p-JNK (sc-6254), JNK (sc-7345), TRAF6 (sc-8409), β-actin (sc-81178)
Cell signaling Tech. (Danvers, MA, USA)	p-p65 (#3033), p-IκBα (#9246), p-IKKα/β (#2697), IKKα (#2682), IKKβ (#2370), p-c-Fos (#5348), c-Jun (#9165), p-p38 (#9215), p38 (#9212), TAK1 (#4505), occludin (#91131), claudin-1 (#13255)
Invitrogen (Carlsbad, CA, USA)	p-TAK1 (#MA5-15073), ZO-1 (#PA5-85256), and MUC2 (#PA5-103083)

Supplementary Table S4. Antibodies used in flow cytometry.

Fluorophore	Antibody
PerCP/Cyaninie5.5	anti-mouse CD3ε, anti-mouse CD11c, anti-mouse Ly6G
PE	anti-mouse CD4, anti-mouse CD11b, anti-mouse NK 1.1
PE/Cyanine7	anti-mouse Ly6C, anti-mouse I-A^b (MHC Ⅱ)
FITC	anti-mouse CD8, anti-mouse CD19, anti-mouse CD25, anti-mouse F4/80, anti-mouse IL-17A
APC	anti-mouse B220, anti-mouse IFN-γ, anti-mouse IL-4
Alexa Fluor® 647	anti-mouse FoxP3

Supplementary figures

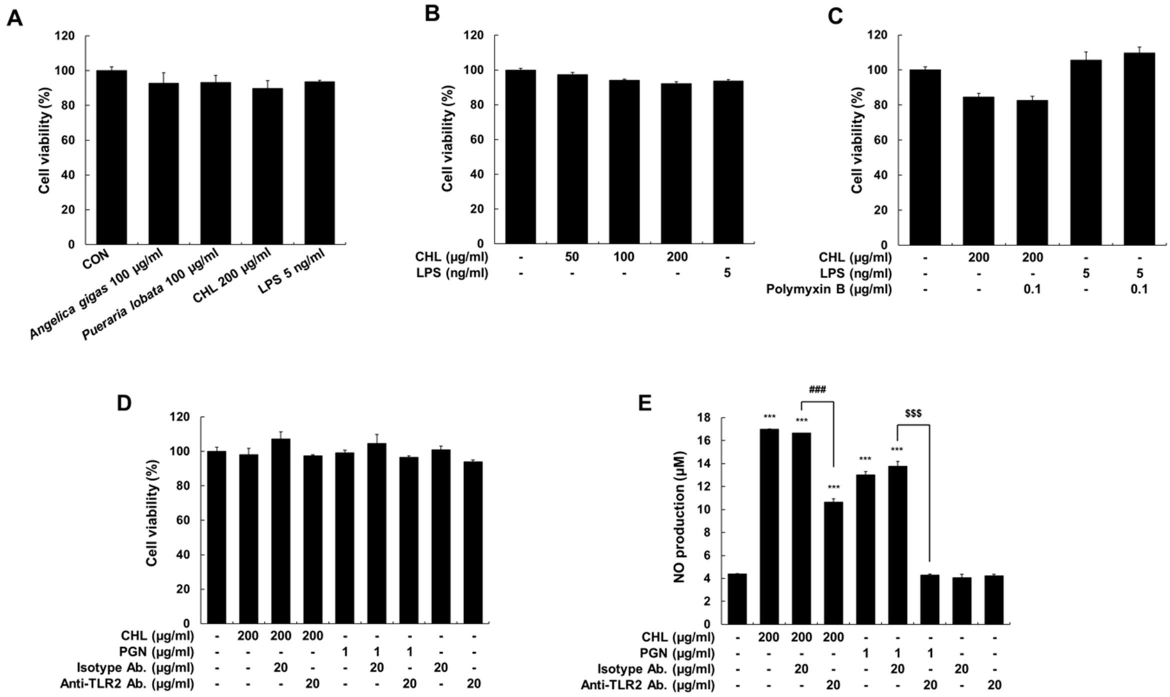

Supplementary Figure S1. Effects of *Angelica gigas*, *Pueraria lobata*, and CHL on cell viability in RAW 264.7 macrophages. (**A**,**B**) RAW 264.7 macrophages were stimulated with *Angelica gigas* (100 μg/ml), *Puerariae Flos* (100 μg/mL), CHL (50, 100, or 200 μg/mL) or LPS (5 ng/mL) for 24 h. (**C**) Cells were pretreated with polymyxin B (0.1 μg/ml) and then stimulated with CHL (200 μg/mL) or LPS (5 ng/mL) for 24 h. (**D**,**E**) Cells were pretreated with isotype or anti-TLR2 antibody (20 μg/mL) and then stimulated with CHL (200 μg/mL) or peptidoglycan (PGN) (1 μg/mL) for 24 h. Data are presented as the means ± SEM of three independent experiments. $^{***}P < 0.001$ vs. CON, $^{\#\#\#}P < 0.001$ vs. isotype antibody plus CHL treated cells, $^{\$\$\$}P < 0.001$ vs. isotype antibody plus PGN treated cells.

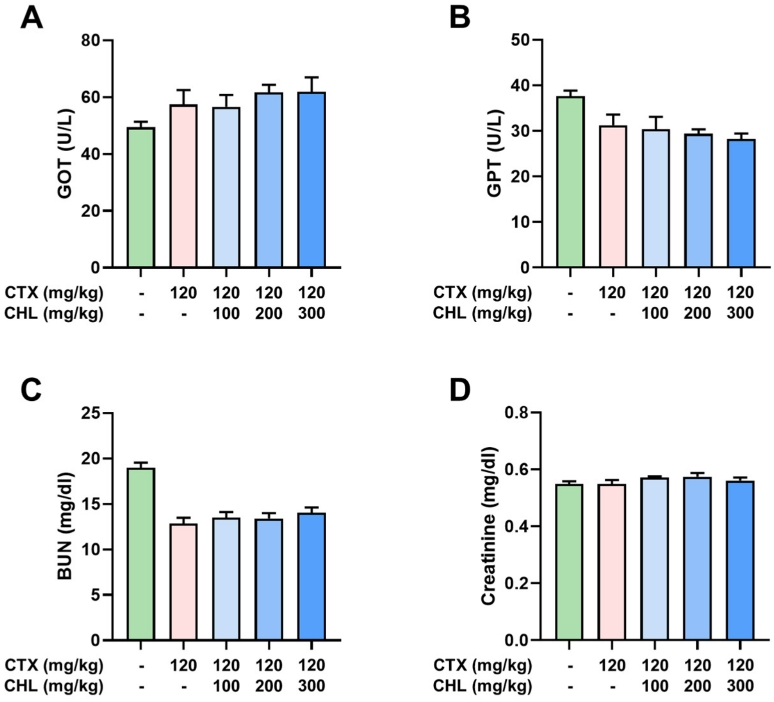

Supplementary Figure S2. Effects of CHL on GOT, GPT, BUN, and creatinine production in cyclophosphamide (CTX)-induced mice. The production of (**A**) GOT, (**B**) GPT, (**C**) BUN, and (**D**) creatinine in the CTX-induced mice plasma. Data are presented as the means ± SEM (*n* = 8).

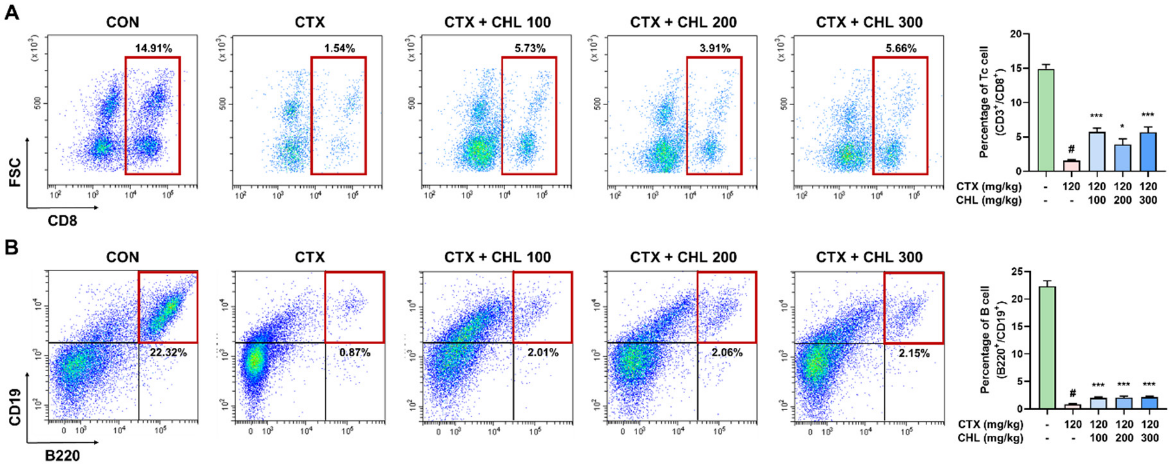

Supplementary Figure S3. Effects of CHL on cytotoxic T cell and B cell populations in CTX-treated mice. (**A**) The population of $CD3^+/CD8^+$ cytotoxic T cells and (**B**) $B220^+/CD19^+$ B cells. Data are presented as the means ± SEM (*n* = 7). $^{\#}P < 0.05$ vs. CON group; $^{*}P < 0.05$, $^{***}P < 0.001$ vs. CTX group.

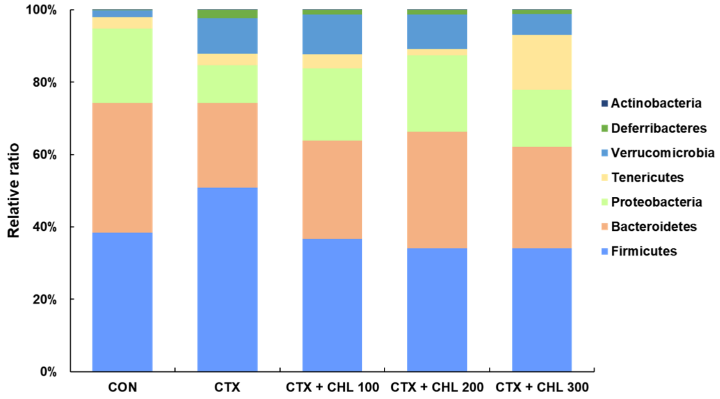

Supplementary Figure S4. Effects of CHL on the relative ratio of gut microbiota composition (phylum) in CTX-induced mice (n=8).

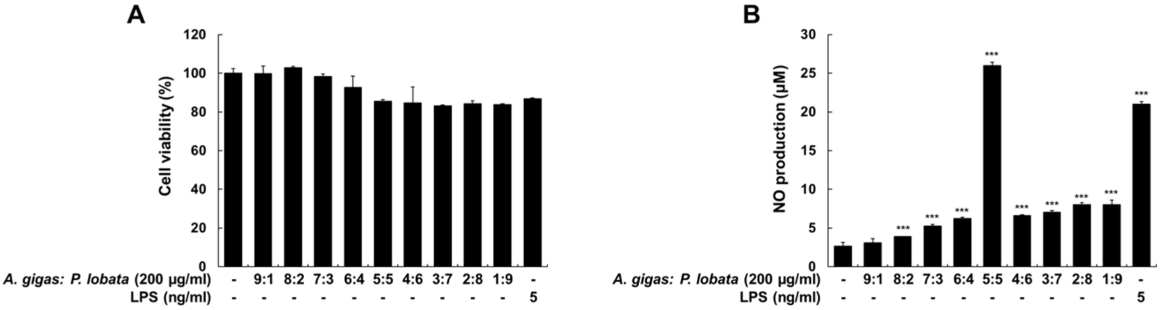

Supplementary Figure S5. Effects of combination ratio of *A. gigas* and *P. lobata* on cell viability and NO production in RAW 264.7 macrophages. (**A**,**B**) RAW 264.7 macrophages were stimulated with *A. gigas*, *P. Flos*, or LPS (5 ng/mL) for 24 h. Data are presented as the means ± SEM of three independent experiments. $^{***}P < 0.001$ vs. CON.

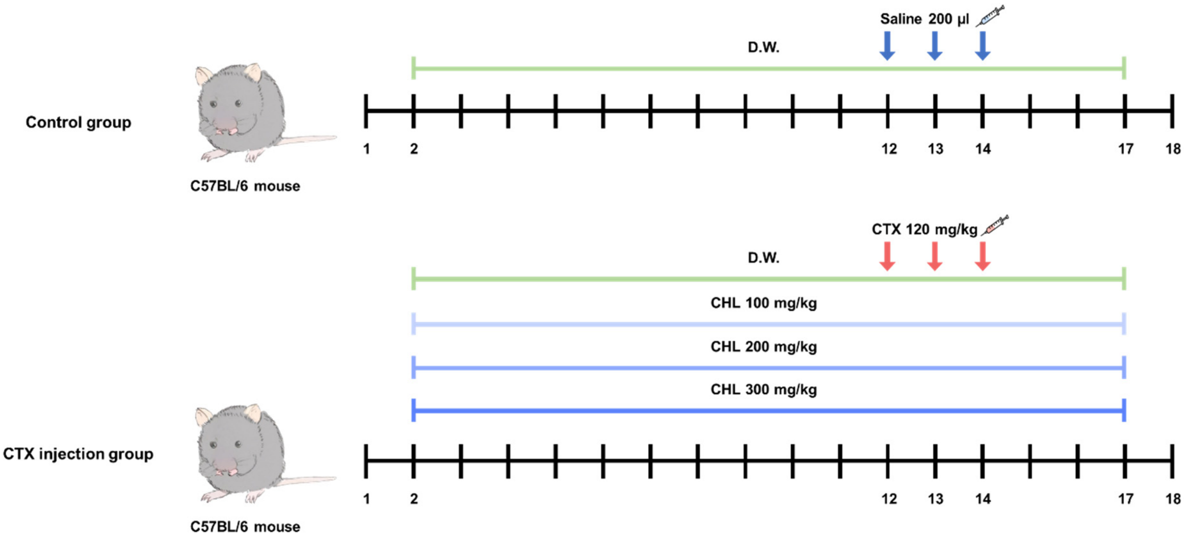

Supplementary Figure S6. Scheme of animal experiments. Establishment of CTX-induced immunosuppressed mice model and oral administration of CHL (100, 200, or 300 mg/kg/day) for 16 days.

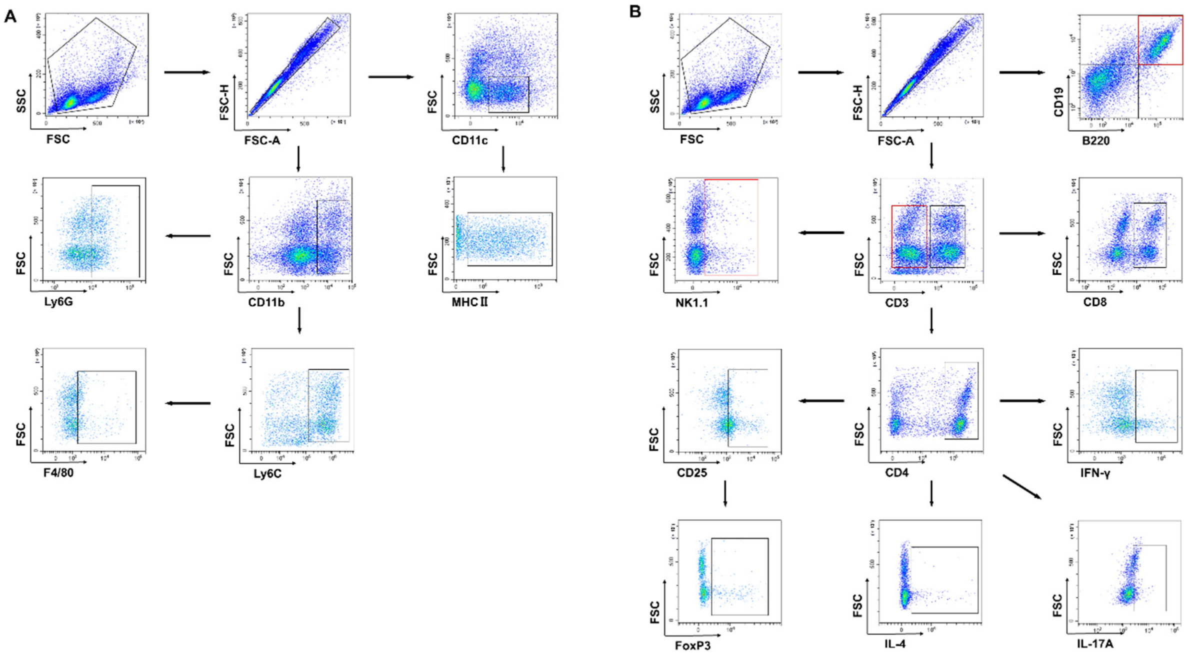

Supplementary Figure S7. Gating methods of immune cells' population in CTX-treated mice. (**A**) Gating of macrophages, neutrophils, dendritic cells, and monocytes, and (**B**) T, Th, Th1, Th2, Th17, Treg, Tc, B, and NK cells.

References

1. S.Y. Jang, S.Y. Kim, H.A. Song, H. Kim, K.S. Chung, J.K. Lee, K.T. Lee, Protective effect of hydrangenol on lipopolysaccharide-induced endotoxemia by suppressing intestinal inflammation, Int Immunopharmacol 125(Pt A) (2023) 111083.
